# Antiplatelet Agents Can Promote Two-Peaked Thrombin Generation in Platelet Rich Plasma: Mechanism and Possible Applications

**DOI:** 10.1371/journal.pone.0055688

**Published:** 2013-02-06

**Authors:** Ivan D. Tarandovskiy, Elena O. Artemenko, Mikhail A. Panteleev, Elena I. Sinauridze, Fazoil I. Ataullakhanov

**Affiliations:** 1 The Laboratory of the Molecular Mechanisms of Hemostasis, the Center for Theoretical Problems of Physicochemical Pharmacology RAS, Moscow, Russia; 2 The Laboratory of Physical Biochemistry of Blood, the National Research Center for Hematology, Moscow, Russia; 3 Department of Physics, Moscow State University, Moscow, Russia; 4 The Laboratory of Biophysics, Federal Research and Clinical Center of Pediatric Hematology, Oncology and Immunology, Moscow, Russia; Institut National de la Santé et de la Recherche Médicale, France

## Abstract

**Background:**

Thrombin generation assay is a convenient and widely used method for analysis of the blood coagulation system status. Thrombin generation curve (TGC) is usually bell-shaped with a single peak, but there are exceptions. In particular, TGC in platelet-rich plasma (PRP) can sometimes have two peaks.

**Objective:**

We sought to understand the mechanism underlying the occurrence of two peaks in the PRP thrombin generation curve.

**Methods:**

Tissue factor-induced thrombin generation in PRP and platelet-poor plasma (PPP) was monitored using continuous measurement of the hydrolysis rate of the thrombin-specific fluorogenic substrate Z-Gly-Gly-Arg-AMC. Expression of phosphatidylserine (PS) and CD62P on the surface of activated platelets was measured by flow cytometry using corresponding fluorescently labeled markers.

**Results:**

The addition of the P_2_Y_12_ receptor antagonist MeS-AMP (160 µM), 83 nM prostaglandin E_1_ (PGE_1_), or 1.6% DMSO to PRP caused the appearance of two peaks in the TGC. The PS exposure after thrombin activation on washed platelets in a suspension supplemented with DMSO, PGE_1_ or MeS-AMP was delayed, which could indicate mechanism of the second peak formation. Supplementation of PRP with 1.6% DMSO plus 830 nM PGE_1_ mediated the disappearance of the second peak and decreased the amplitude of the first peak. Increasing the platelet concentration in the PRP promoted the consolidation of the two peaks into one.

**Conclusions:**

Procoagulant tenase and prothrombinase complexes in PRP assemble on phospholipid surfaces containing PS of two types - plasma lipoproteins and the surface of activated platelets. Thrombin generation in the PRP can be two-peaked. The second peak appears in the presence of platelet antagonists as a result of delayed PS expression on platelets, which leads to delayed assembly of the membrane-dependent procoagulant complexes and a second wave of thrombin generation.

## Introduction

The thrombin generation test (TGT) is one of the most informative and popular blood coagulation assays used for both research and diagnostic purposes. This simple technique for measuring thrombin concentration in clotting plasma was presented by H.C. Hemker [1]–[3]. In this method, a thrombin-specific fluorogenic substrate is added to the plasma before triggering coagulation. Thrombin generation curve (TGC) is usually bell-shaped and therefore has four main features. The first is a lag-time, during which the thrombin concentration increases to 5 nM. Then, the rate of thrombin generation (TG) increases abruptly, and thrombin concentration increases, reaches a peak, and begins to decrease to zero. The second and the third features are described by the time and amplitude of the thrombin peak. The most frequently measured parameter is the endogenous thrombin potential (ETP), which is equal to the area under the TGC.

TGT can be used to investigate coagulation under different conditions. Initially, this test was used to explore coagulation in platelet poor plasma (PPP). However, researchers currently can use platelet rich plasma (PRP) in the TGT to investigate platelet contribution to thrombin generation [1], [4]–[6].

TG in PRP decreases in the presence of antiplatelet agents [4]. On the contrary, platelet activators increase TG [4], [5]. This test is also used in clinical investigations of hemophilia [7], [8], thrombosis [9], von Willebrand's disease [10], anticoagulant and antiplatelet therapy [11], [12]. Thus, measuring TG in PRP has numerous clinical applications.

In the published literature, TGCs with different shapes have been obtained under various conditions. The majority of PPP-derived TGCs are smooth with a single thrombin peak. However, in PRP-TGCs and under other conditions, a second peak (or “shoulder”) (see, for example, [4]) can also be present. This second peak suggests the existence of two periods of accelerated TG in PRP. The study of mechanisms leading to two-peaked TGCs formation may be helpful for better understanding of processes, which take a place in blood coagulation under different conditions. However, to present day the shapes of TGCs have been addressed only in one study [13] where some problems of approximation of TGCs are discussed.

The formation of procoagulant complexes during blood coagulation leads to the acceleration of TG. The assembly of procoagulant complexes takes place both on the surface of plasma lipoproteins and on the surface of activated platelets expressing phosphatidylserine (PS) [14], [15]. The time to the maximal thrombin concentration depends on the rate of complex formation. In the case of platelets, this formation is determined by the rate of platelet activation. We hypothesized that, in PRP, the rates of lipoprotein- and platelet-mediated procoagulant complex formation may be different. Platelet activation inhibitors may reduce the rate of platelet activation [16] and delay or decrease the expression of PS on their membranes [17], [18]. In this case, the formation of the second peak may be the result of these late activated platelets.

## Materials and Methods

### Solutions

Buffer A for platelet storage contained 150 mM NaCl, 2.7 mM KCl, 1 mM MgCl_2_, 0.4 mM NaH_2_PO_4_, 20 mM HEPES, 5 mM glucose and 0.5% BSA (pH 6.5 or 7.4). All reagents were from Sigma-Aldrich (St. Louis, MO, USA). Buffer B was used to adjust the plasma pH (145 mM NaCl, 750 mM HEPES, pH 7.4). This buffer was added to a sample to obtain final HEPES concentration equal to 24 mM. Buffer C was used for TGT and contained 20 mM HEPES, 145 mM NaCl, pH 7.5. Buffer D was the same as buffer C, but it also contained 100 mM CaCl_2_ (Sigma-Aldrich, St. Louis, MO, USA). Coagulation was triggered by an activator, which was a solution of tissue factor (TF, Innovin, Dade-Behring, Marburg, Germany) in buffer D. The final TF concentration in the plasma sample was equal to 0.5, 2 or 4 pM. The Actichrome TF activity assay (American Diagnostica, Stamford, CT, USA) was used to determine the TF activity. The fluorogenic substrate Z-Gly-Gly-Arg-AMC (Bachem, Bubendorf, Switzerland) was stored at −20°C as a solution in DMSO (Sigma-Aldrich, St. Louis, MO, USA) at concentrations of 200 mM or 25 mM. These solutions were diluted to 2.5 mM with buffer C before each experiment.

### Blood collection

All donors were healthy volunteers who were free of medication. Blood was collected by venipuncture and placed into a test tube with 3.8% sodium citrate (pH 5.5). The volume ratio between blood and citrate was 9∶1.

### Ethics statement

This work was part of a multicenter study called “Investigation of the Pathologic and Physiologic Mechanisms of Hemostasis” conducted by the Center for Theoretical Problems of Physicochemical Pharmacology of the Russian Academy of Sciences and the National Center for Hematology. The design of the research was developed following the recommendations of the Interinstitutional Committee for Ethics of Medical and Pharmaceutical Institutions Association. The research design of this part of the study and informed consent forms were approved by the Ethics Committee of the Center for Theoretical Problems of Physicochemical Pharmacology of the Russian Academy of Sciences. Each participant signed the written form of informed consent before blood collection.

### Plasma preparation

To prepare the PPP, blood was centrifuged for 15 min at 1600 g, and 2/3 of the plasma supernatant was collected. PRP was prepared by blood centrifugation for 5 min at 100 g. To reach the required platelet concentration, the PRP was diluted with autologous PPP.

### Platelet isolation

Prostaglandin E_1_ (PGE_1_) (830 nM final concentration, MP Biochemicals, Solon, Ohio, USA) and 0.1 unit/ml apyrase (Sigma-Aldrich, St. Louis, MO, USA) were added to the blood immediately after collection, and the PRP was prepared. Sodium citrate (3.8%, pH 5.5) was added to the PRP at a volume ratio of PRP∶citrate = 3∶1. This mixture was centrifuged at 400 g for 5 min. The sediment was then resuspended in 300 µl of buffer A at pH 6.5. Gel-filtered platelets were prepared as previously described [19].

### Thrombin generation assay

The plasma was mixed with substrate solution at a volume ratio of 4∶1. Then, 20 µl of buffer B was added per 500 µl of this mixture for pH stabilization, and it was placed into the wells of a flat-bottom 96-well plate. Each well contained 100 µl of the mixture. Coagulation was triggered with 20 µl of activator (see *Solutions*) per well. The final fluorogenic substrate concentration was 400.6 µM.

For calibration of fluorescence signal, two types of wells with non-clotting plasma were prepared. The first type contained 20 µl of buffer C instead of the activator to obtain the background fluorescence level. The second one (calibration wells) contained 2 µl of AMC (Sigma-Aldrich, St. Louis, MO, USA) solution in DMSO and 18 µl of buffer C instead of the activator. The final AMC concentration in these wells was 8 µM. Fluorescence was continuously measured at 37°C using an Appliskan Multimode Microplate Reader (Thermo Scientific, Helsinki, Finland) (λ_exc_ = 355 nm, λ_em_ = 460 nm). The PRP was not stored for more than 1 hour before use. All results are reported as the averaged readings of duplicated wells.

Data analysis was carried out using Origin 6.0 or 8.0 software (Microcal Software, Northampton, MA, USA). The background fluorescence was subtracted from the signal of fluorescence in all of the wells. To calculate the AMC concentration, we used the appropriate calibration wells and considered the nonlinear fluorescence dependence on AMC concentration (inner filter effect [2]). The thrombin curve was obtained by differentiation of the time-dependent AMC curve (after its smoothing) and calculation of the thrombin concentrations using the previously measured kinetic constants for a given fluorescence substrate (K_M_ = 156 µM; k_cat_ = 46 min^−1^). The substrate concentration corresponding to each time point was calculated by subtracting the concentration of produced AMC from the initial substrate concentration. So, we took into account the substrate consumption in our technique. The contribution of α_2_-macroglobulin [1]–[3] was subtracted using a specially designed algorithm realized in Origin software as described previously [20].

### Determination of the experimental error in TGT

The method of numerical differentiation for the calculation of the thrombin concentration is characterized by an experimental error that increases with increasing AMC concentrations. To evaluate the expected error in each part of the TGC, we performed four identical TG tests with PRP (100·10^3^ platelets per µl) from a single healthy donor with activation by 2 pM of TF (Fig. S1A in File S1). The standard deviation (SD) for the thrombin concentration at each point of the averaged curve was calculated from these four TGCs. Then, the SD dependences on time and on the AMC concentration were calculated (Fig. S1B, S1C in File S1). In this way, we determined that, during the first 50 min (or when the AMC concentration was lower than 100 µM) the maximal SD was equal to 2.6 nM (Fig. S1D in File S1). During this period of time, the thrombin concentration was much more than maximal SD. During the next 40 min, the maximal SD was equal to 4 nM, which was comparable to the thrombin concentration obtained in this period. Over 90 min after triggering coagulation, the SD for thrombin measurement significantly exceeded the thrombin concentration itself. Therefore, we used 90 min as the period of measurements in our experiments.

### Flow cytometric assay of platelet activation kinetics

Samples comprising 100 µl of the suspension of gel-filtered platelets (200·10^3^ platelets per µl) were supplemented with 1 µl of annexin V labeled with R-phycoerythrin (annexin V-RPE, Invitrogen, Carlsbad, CA, USA), 1 µl of anti-CD62P labeled with fluorescein isothiocyanate (anti-CD62P-FITC, BD biosciences, Franklin Lakes, NJ, USA) and 5 µl of buffer D. Then, 100 µl of human α-thrombin solution (Haematologic Technologies, Essex Junction, VT, USA, 70 nM in buffer A) was added to platelets. In some cases, the thrombin solution contained DMSO, PGE_1_ or the P_2_Y_12_ antagonist 2-methyltioadenosine-5′-O-monophosphate (MeS-AMP, [21], Biolog inc., Hayward, CA, USA). To measure the PS and CD62P expressions, a FACSCalibur flow cytometer (BD Biosciences, Franklin Lakes, NJ, USA) was used. Each sample was diluted ten times in buffer A with 2.5 mM CaCl_2_ before measurement. The cytometric measurements were carried out before and 5, 10, 15 and 25 min after thrombin addition.

The data analysis was performed with Flowing Software created by Perttu Tehro (Turku Centre for Biotechnology, Turku, Finland). The region of platelets in the dot plot was identified as shown in Figure S2 in File S1. As a result, the mean annexin V-RPE fluorescence and the percentage of CD62P-positive platelets in the platelet region were calculated.

## Results

### MeS-AMP, DMSO and low doses of PGE_1_ induce the appearance of the second peak of the PRP TGC

**Figure 1:**
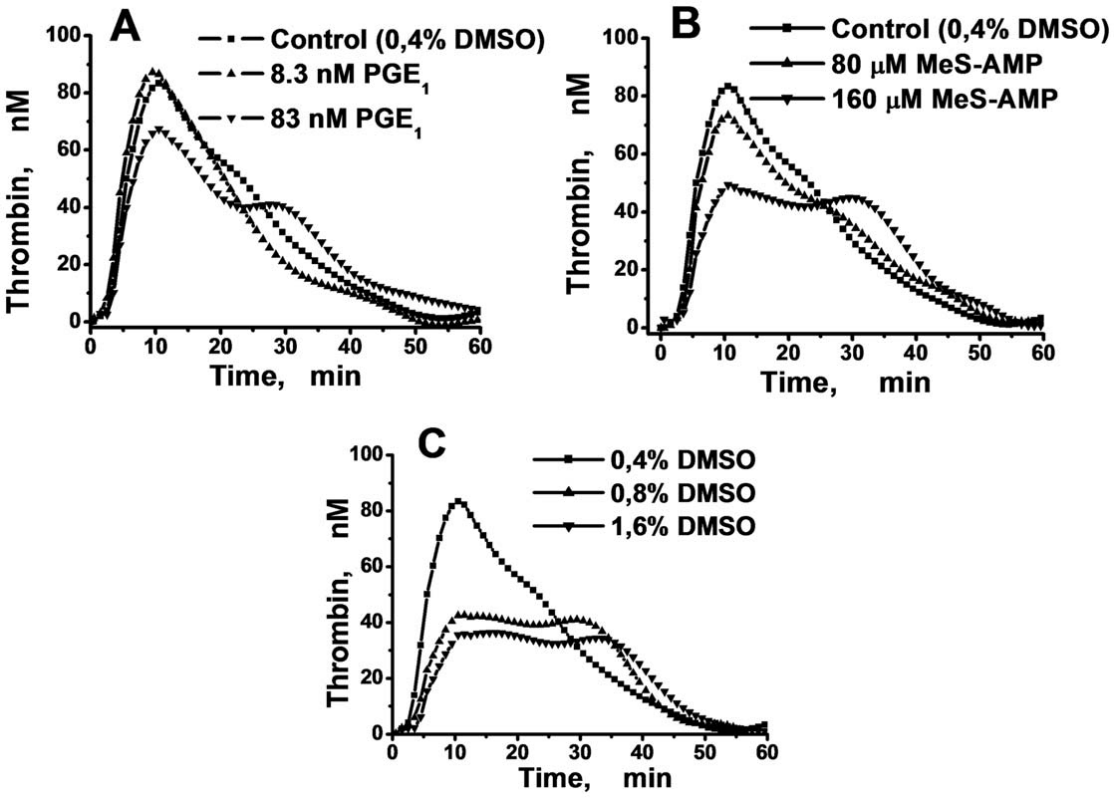
The formation of the second peak in PRP TGCs mediated by PGE_1_, MeS-AMP, or DMSO. Thrombin generation in the PRP of a healthy volunteer (the final platelet concentration in the experimental wells was equal to 100·10^3^ µl^−1^) was triggered by 2 pM of TF in the presence of different concentrations of PGE_1_ (A), MeS-AMP (B), or DMSO (C).

In our experiments, the addition of 83 nM of PGE_1_ into PRP resulted in the appearance of the second TGC peak and reduced the amplitude of the first peak (Fig. 1A). Similar results were obtained using MeS-AMP (Fig. 1B). It is important to mention that the effect of MeS-AMP varied in different plasma samples. The amplitude of the first peak could be unchanged, increased or decreased in the presence of MeS-AMP in different samples, but in every plasma sample, this platelet antagonist induced two peaks in the TGC (Fig. S3 in File S1).

Although DMSO is an artificial compound with a poorly understood mechanism of action, we cannot avoid its presence in the plasma during the measurement of TG because DMSO is a solvent for the fluorogenic substrate. It was shown previously [22], [23] that DMSO is able to inhibit platelet activation. Therefore, we tested whether increasing the DMSO concentration could lead to the formation of the second peak. Our preliminary results showed that DMSO did not impact the thrombin activity at the concentrations that we used (data are not shown). The presence of DMSO induced the formation of the second peak (Fig. 1C). The magnitude of the effect depended on the plasma sample. In some cases, we observed two peaks on the TGC even at a DMSO concentration as low as 0.4% (Fig. S4 in File S1). It is the basic concentration, which is widely used in TGT [4].

Thus it was shown that the effects of PGE_1_, MeS-AMP, and DMSO on TG were qualitatively similar. All of these compounds provoked the appearance of the second peak. At the same time, these inhibitors poorly influenced the total ETP values, which were changed by no more than 20% (Fig. S5 in File S1).

### A strong increase in the PGE_1_ concentration reduces the second TGC peak in a dose-dependent manner

To demonstrate that the formation of the second TGC peak was related to the presence of slowly activated platelets, we attempted to gradually block platelet activation until complete inhibition was achieved by increasing the PGE_1_ concentrations. We used DMSO (1.6%) to produce two peaks on the PRP-TGC. Then different concentrations of PGE_1_ were added to PRP samples.

**Figure 2:**
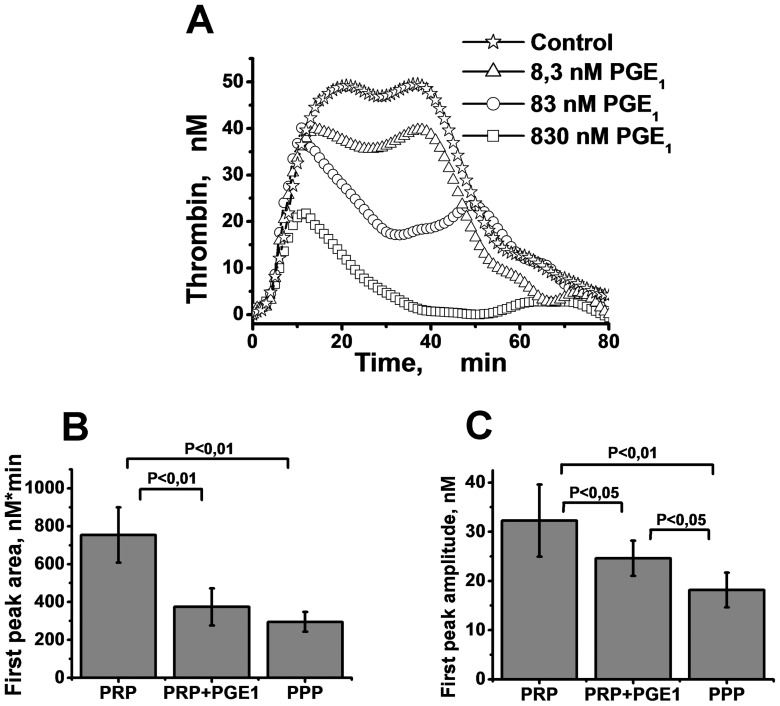
Influence of PGE_1_ on TG in PRP. A: TGCs in PRP supplemented with 1.6% DMSO in the presence of different PGE_1_ concentrations. PRP without PGE_1_ was used as a control. B, C: The mean values of areas (B) and amplitudes (C) of the first peak in PRP, PRP with 830 nM PGE_1_ addition, and PPP. Coagulation was activated with 2 pM of TF. The platelet concentration was 100·10^3^ platelets per µl. Mean values and SD are presented; n = 18, 8, and 7 for PRP, PRP+PGE_1_, and PPP, correspondingly. Student's t-test with P values equal to 0.05 and 0.01 was used to obtain statistics. The difference is not significant in all the bars where P-value is not presented.

The results obtained are presented in Figure 2. The amplitude of the second peak was reduced, and the time to the second peak was prolonged in PRP containing 1.6% DMSO depending on the PGE_1_ concentration (Fig. 2A). In several cases, 83 nM of PGE_1_ totally blocked the formation of the second peak. The addition of a high dose of PGE_1_ (830 nM) always led to the disappearance of the second peak. PGE_1_ also affected the amplitude of the first peak, but less dramatically (Fig. 2B, 2C). The method to obtain the peak amplitude is described below in the section *Analysis of the parameters for each peak in the thrombin generation curve*.

### PGE_1_, DMSO and MeS-AMP decrease the platelet PS exposure rate, but they do not influence P-selectin expression

**Figure 3:**
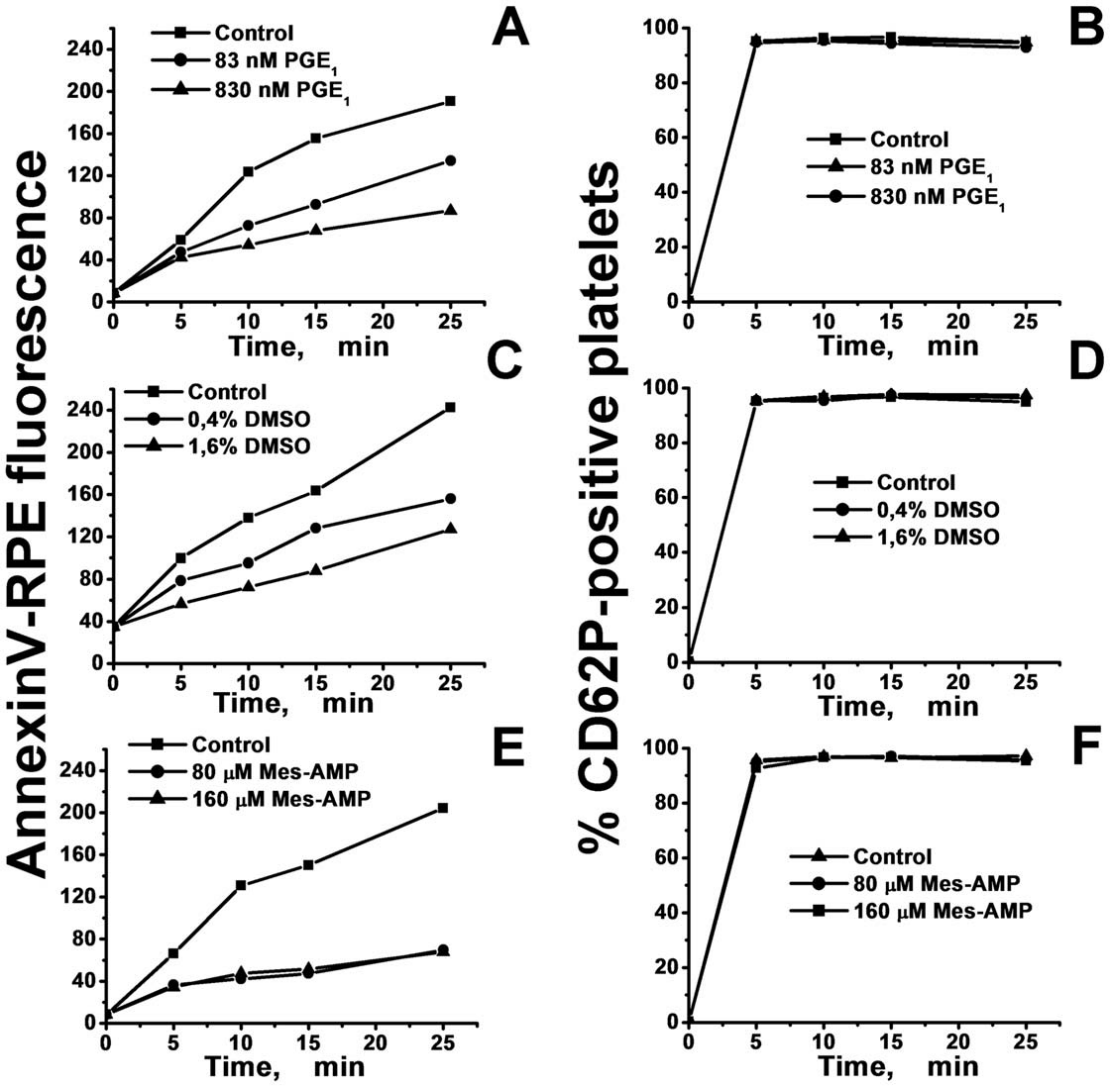
Kinetics of platelet activation in the presence of PGE_1_, DMSO, and MeS-AMP. A suspension of gel-filtered platelets (100·10^3^ cells per µl) labeled with annexin V-RPE and anti-CD62P-FITC was activated with human α-thrombin solution (35 nM final concentration) containing PGE_1_ (A,B), DMSO (C,D), or MeS-AMP (E,F). The mean RPE (A,C,E) fluorescence and the percentage of CD62P-positive platelets (B,D,F) after different incubation times is presented.

The ability of PGE_1_, DMSO, and MeS-AMP to inhibit activation of washed platelets in suspension was studied by flow cytometry. All of these agents were able to reduce the rate of the mean annexin V-RPE fluorescence increase in freshly isolated, gel-filtered platelets after their activation by thrombin (35 nM) (Fig. 3A, 3C, 3E). In contrast, these compounds did not affect the percentage of CD62P-positive platelets (Fig. 3B, 3D, 3F). These results indicate that, under the selected activation conditions, PGE_1_, DMSO, and MeS-AMP slow the rate of exposure of PS on platelet membranes, but they do not influence P-selectin expression or α-granules secretion. Additionally, this result suggests that the second peak in the PRP-TGC can be mediated by delayed platelet PS expression.

Although PGE_1_ is known to inhibit platelet CD62P release [24], this effect is dependent on the degree of activation. For example, if a suspension of platelets is activated by weak activator such as thromboxane A_2_ mimetic [24], addition of PGE_1_ can reduce P-selectin expression. In our work we used thrombin, which is much stronger activator than thromboxane A_2_. The thrombin concentration in our cytometric experiments (35 nM) was chosen to be comparable to that observed in TGT. We hypothesized that at these conditions addition of PGE_1_ will not affect CD62P release. To test this suggestion, we performed additional experiments, where platelet suspensions were activated by different thrombin concentrations (Fig. S6 in File S1). The result presented in Figure S6 in File S1 shows that PGE_1_ affects CD62P release only when thrombin concentration is much lower than one we used in our main experiments.

### Increasing the platelet concentration can transform two peaks on the TGC into one

**Figure 4:**
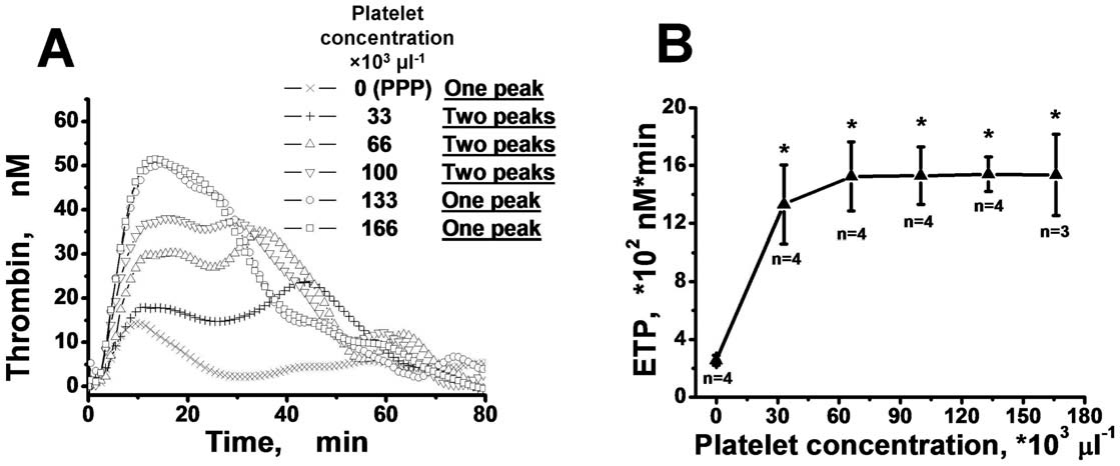
Increasing platelet concentration causes the integration of two peaks into one. A: Curves of thrombin generation obtained in a typical experiment with the plasma of a healthy donor containing 1.6% of DMSO and different concentrations of platelets. Coagulation was triggered with 2 pM of TF. B: The dependence of the mean ETP values and SD on platelet concentration for healthy donors (n – number of donors; * - difference between PRP and PPP is significant (Student's t-test, P<0.01)).

As indicated above, total inhibition of platelet activation leads to the disappearance of the second peak in TGC. We decided to study whether platelet concentrations could somehow affect the “two peak” phenomenon. In Figure 4A, TGCs measured using different platelet concentrations are presented. In all of these experiments, addition of 1.6% DMSO was used to generate two-peaked TGCs. We observed that the time to the appearance of the second peak decreased with the increase of the platelet concentration. If the platelet concentration was 133·10^3^ or 166·10^3^ platelets per µl, the majority of the TGCs had one peak (Fig. S7A–D in File S1). The peaks were defined using the method described below. Nevertheless, the platelet concentration in PRP did not have a considerable impact on the ETP value, while ETP in PPP was significantly lower than ETP in PRP (Fig. 4B).

### Analysis of the parameters for each peak in the thrombin generation curve

**Figure 5:**
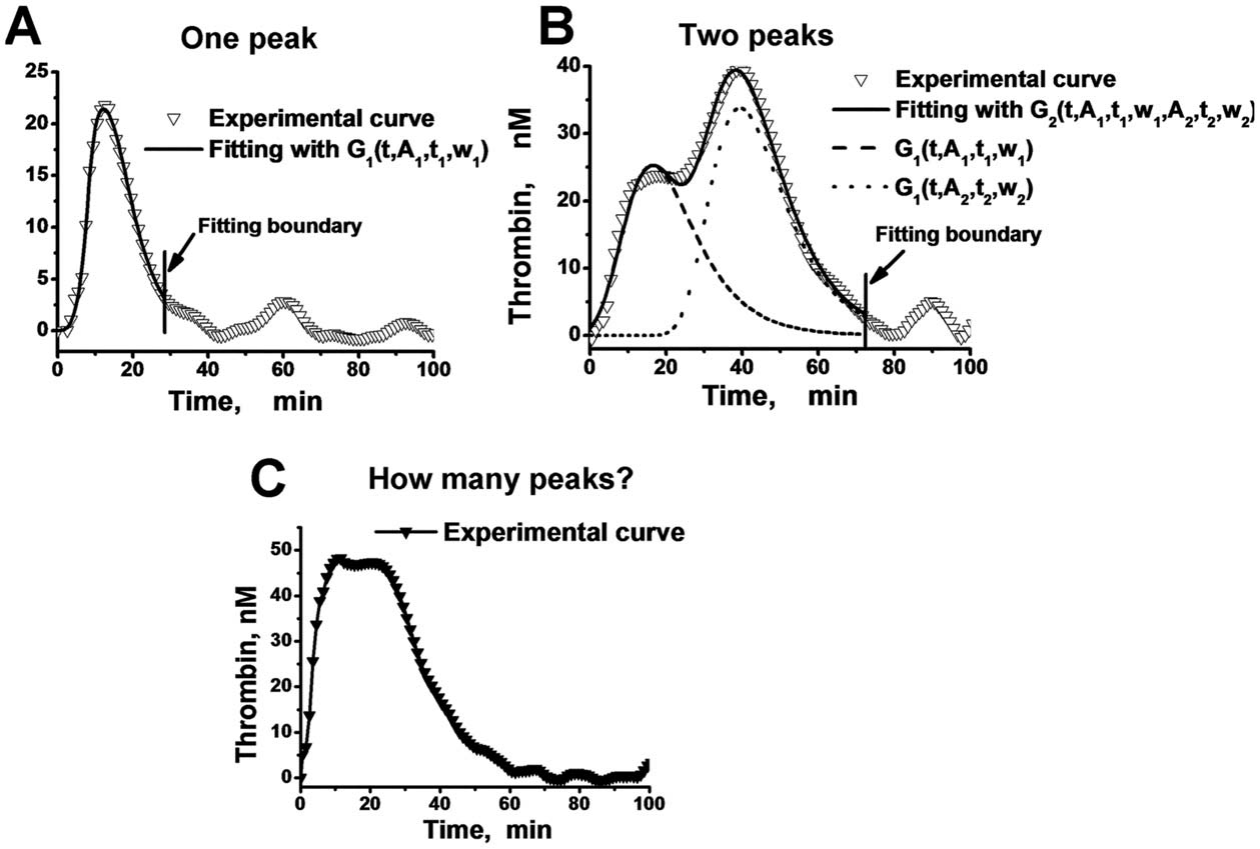
Analysis of thrombin generation peak parameters. For approximation of TGCs with one peak, a type I Gumbel's distribution function was used (A). The TGCs with two peaks were described by superposition of two extreme distribution Gumbel's functions (B). In some cases, it was impossible to determine how many peaks the TGC contained (C). Fitting was performed until the thrombin concentration began to be comparable with the experimental error (see Fig. S1 in File S1).

Increasing the number of results obtained with different plasmas under different conditions (different concentrations of platelets, TF, and inhibitors) required creating a method for the analysis of the peak parameters. Furthermore, in some cases there was a problem with the definition of the number of peaks that a curve contains. For the analysis of TGCs with one thrombin peak (mostly samples of PPP or PRP with high PGE_1_ doses), we used fitting with Gumbel's type I extreme distribution function [25] (1):
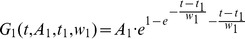
(1)where *t*, *A_1_*, *t_1_*, and *w_1_* correspond to time, peak amplitude, time to peak achievement, and peak half-width, respectively (Fig. 5A). This function approximated our one-peak curves with a correlation coefficient R_1_>0.91. For analysis of the TGCs containing two peaks (the majority of our PRP samples), the superposition of two distribution Gumbel's functions (2) was used (Fig. 5B):
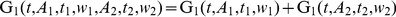
(2)However, there were many curves for which we could not visually determine the number of peaks (Fig. 5C). So, we needed some mathematical criterion to determine how many peaks the curve contains. The correlation coefficient (R) was not sufficient for this. It was always larger for G_2_ fitting (R_2_) than for G_1_ fitting (R_1_) because of the greater number of parameters, which allowed better approximation of the experimental curve by the correlation function. Thereby, G_2_-fitting was better even for obviously one-peaked curves. However, the R_2_/R_1_ ratio was quite different between the cases with clearly determined numbers of peaks (one or two). In Table 1, the ranges of these ratios for one- and two-peaked TGCs are presented. We can see that the curves with one peak have the R_2_/R_1_ less than 1.06, but this ratio is more than 1.07 for TGCs containing two peaks. Therefore, to designate an experimental TGC as a one- or two-peaked curve (see Fig. 5), the value of the R_2_/R_1_ ratio was calculated. If it was in the one-peak range (Table 1), we assumed that the TGC had one peak. If R_2_/R_1_ was in the two-peak range, we assumed that there were two peaks on the curve. If the R_2_/R_1_ ratio value was between 1.06 and 1.07, we stated that the number of peaks could not be determined reliably.

**Table 1:** Criteria for determination of the quantity of TGC peaks*.

Number of peaks	Number of curves	R_2_ range	R_1_ range	R_2_/R_1_ range
1	19	0.915–0.998	0.912–0.994	1.001–1.056
2	30	0.909–0.996	0.574–0.927	1.072–1.680

* The first row displays the ranges of coefficients of correlation between the TGC and the two- or one-peak fitting (R_2_ and R_1_, respectively) and the ranges of the R_2_/R_1_ ratio for 19 TGCs known to contain one peak. The second row shows the same parameters for 30 curves known to contain two peaks.

Analysis of all parameters related to thrombin generation was carried out for all of the obtained TGCs. The majority of the TGCs obtained from the PPP samples had one thrombin concentration peak. The PRP samples often had two thrombin concentration peaks. The results for two-peaked TGCs obtained after activation of thrombin generation by 2 pM of TF are presented in Figure S8 in File S1. Similar results were obtained with 0.5 and 4 pM TF (data are not shown).

The areas under the first and the second peak in our experiments with PRP supplemented with 1.6% DMSO were approximately identical (Fig. S8A in File S1). None of the parameters of the two peaks differed significantly with the exception of the time to the first and the second peak (Fig. S8C in File S1).

It was also shown (Fig. 2B, 2C, Fig. S9 in File S1) that the peak amplitudes in the PPP samples and in PRP samples supplemented with PGE_1_ are significantly decreased compared to PRP alone. However, the time to peak and the half-width of the first peak were not significantly different for the PRP and PPP samples, but were significantly decreased compared to PRP after addition of PGE_1_ to PRP (Fig. S9 in File S1).

## Discussion

It was shown previously [4], [5] that TGCs sometimes present several regions of accelerated thrombin formation. This fact has typically been ignored by researchers, but we proposed that, in these cases, the thrombin generation curve may have two peaks that are not completely separated. The results of our analysis of curves shape correspond well with result of the work [13] where also fitting of TGCs was made. In this article authors used function (W-function) which was close to the Gumbel curve used in our research. Similarly to Gumbel function, W-function approximated PPP TGCs quite well, but PRP TGCs could be fitted only with superposition of two W-functions.

**Figure 6:**
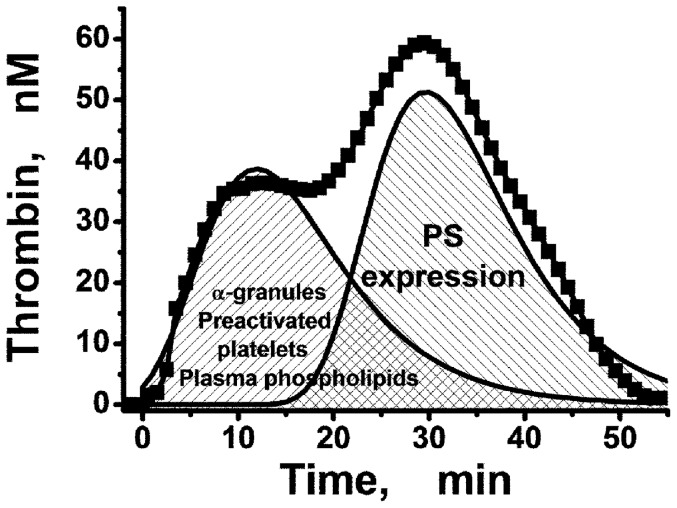
Contribution of phospholipid surfaces to the first and the second thrombin generation peaks. Plasma phospholipids, α-granules and preactivated platelets form the first peak. PS expression on the platelets' surface can mediate the second one formation.

There are different types of phospholipid surfaces on which procoagulant complexes of plasma coagulation factors can be assembled (Fig. 6). In PPP, the majority of these surfaces are presented by plasma lipoproteins containing phospholipids including PS. PPP contains a low concentration of platelets (1000–2000 µl^−1^). So, the contribution of these cells to the assembly of procoagulant complexes after clotting activation may be low. In PPP, thrombin generation occurs mainly on the surface of plasma phospholipids. In this situation, the majority of the procoagulant complexes begin assembling at the same time, because all types of presented phospholipid surfaces are ready for coagulation (Fig. 6) and the TGC has only one thrombin concentration peak.

However, this is not the case for PRP. The surface of the platelets becomes procoagulant after the expression of PS as a result of platelet activation. The process of this activation may be delayed if platelet activation inhibitors are present in the plasma. Therefore, our hypothesis is as follows: in platelet-rich plasma, procoagulant prothrombinase and tenase complexes may be assembled on two types surfaces - on plasma lipoproteins, where complexes form with kinetics similar to those in PPP, as well as on surface of activated platelets. Additionally, the release of α-granules and the low level of preactivated platelets in PRP can increase the rate of TG mediated by plasma phospholipids. Formation of the complexes on the surface of activated platelets may be prolonged by various inhibitors of platelet activation. In this case, the TGC may have two different thrombin concentration peaks as we have shown in Figure 1.

We suppose that the normal plasma kinetics of procoagulant complex formation on plasma phospholipids and on the surface of activated platelets are similar, which explains the presence of a single thrombin peak, even in PRP, if the sample does not contain any platelet inhibitors, for example, a high concentration of DMSO. At the same time, it was shown in this study that the presence of inhibitors of platelet activation could at first induce the appearance of the second thrombin peak in the TGC (Fig. 1) and then could completely block that peak at higher inhibitor concentrations (Fig. 2).

The measurement of the kinetics of PS expression and α-granules secretion was carried out in suspensions of washed platelets in the presence of different concentrations of these inhibitors to investigate the possible mechanism of their action (Fig. 3). As a result, it was established that all of these effectors slowed down PS expression, but they did not affect secretion of α-granules, as measured by the expression of CD62P on the surface of the platelets after their activation by high enough thrombin concentration. This result confirms our hypothesis that the appearance of the second peak on the TGC is a result of delayed expression of PS on the platelet membrane due to the presence of platelet inhibitors. In favor of this conclusion, our inhibitors did not decrease the ETP values significantly, but rather they only led to the formation of two-peaked TGCs (Fig. S5 in File S1). The increase of the platelet concentration could compensate for the inhibitor's effects (Fig. 4 in File S1). In these experiments, the ETP increased with increasing platelet concentrations during the transition from PPP to PRP, and the two thrombin peaks obtained in the PRP supplemented with 1.6% DMSO at a low platelet concentration (33·10^3^ µl^−1^) gradually transformed to one peak at higher platelet concentrations (166·10^3^ µl^−1^). There are two possible explanations for this observation: a) platelets become activated faster at higher cell concentrations because of autocatalytic activation processes; b) the surface and intracellular concentration of the inhibitor decreases at higher cell concentrations as a result of increased surface area. However, the total ETP value weakly depended on the platelet concentration in the region of (33–166)·10^3^ µl^−1^, which is consistent with other studies [4], [20], [26].

In our study, we used DMSO, PGE_1_ and MeS-AMP as the inhibitors of platelets in the concentrations, which are used in clinical and laboratory practice. DMSO can be present in patients during therapy with some drugs, or it can be used as anti-inflammatory and anesthetic agent [27]. Also DMSO is used in cell cryopreservation, and, therefore, in stem cell transplantation [28]–[30]. The blood concentration of this agent after intravenous infusion can be estimated by dividing the total volume of DMSO by 4–5 liters of blood. Thereby, we can calculate that DMSO concentration in the blood of the patient can be equal to 0.2–0.4% [28], [29], but sometimes can reach 0.56% [30] and even 1.2% [29], [31]. The mechanism of DMSO action is poorly understood. In the literature, it is mentioned that DMSO can impair platelet adherence and aggregation [22], possibly through cyclooxygenase I [23]. Moreover, the presence of DMSO is essential for ETP measurement in PRP, as it is necessary for the preparation of a corresponding fluorogenic substrate solution, and the concentrations of DMSO used in TGT are able to affect platelet hemostasis (see Fig. S4 in File S1). This effect of DMSO can strongly influence on the results of TGT in PRP, especially, during the exploration of platelet disorders with thrombocytopenia.

PGE_1_ infusions are used to prevent thrombosis development [32]–[34]. Blood concentrations of this agent can be estimated from 10 to 100 nM, but in some cases, they can be much lower [32]. In one work, PGE_1_ concentrations in plasma after intravenous infusion were measured [34]. They were equal to 0.04 nM. But PGE_1_ bound to cell membranes was not included in this result, so the total blood concentration of PGE_1_ could be much higher. The influence of high doses of this compound on TG was investigated previously [4]. In our work, we used the concentrations which are closer to the doses used in clinical practice and are known to inhibit platelet aggregation [35], [36].

The drug clopidogrel (Plavix) is widely used in clinical practice and is a P_2_Y_12_ antagonist. The influence of this receptor on TG was shown previously [4], [5]. However, MeS-AMP was only investigated regarding the inhibition of platelet aggregation in vitro [37], [38] and in regard to its ability to prevent thrombus formation in various models under different conditions of blood flow [39]. This compound is used only for laboratory investigations in concentrations we used in our study. We show that though this compound is able to inhibit platelet PS expression, its effects on platelet-dependent TG can vary (Fig. S3 in File S1). This fact allows us to propose that MeS-AMP may also affect TG through additional undefined mechanisms.

In our research, we used our modification of original technique to explore TG. This method differs from widely used one described by H.C. Hemker [1], [2]. We did not use the calibrator and the special software, but we took into account all possible problems which were discussed by the developers of this assay, such as inner filter effect, influence of thrombin-α_2_-macroglobulin complex and fluorogenic substrate consumption. Our method is rather simple and does not need the calibrator and any special software, which are used in Hemker's technique.

Thus, we found that TG in PRP consisted of two parts. The first one is primarily dependent on plasma phospholipids, and the second part is mediated by the expression of PS on the platelet membrane during platelet activation. In two-peaked TGC phospholipids of plasma induce an “early” peak of thrombin in TGC, whereas prolonged expression of PS on the activated platelets in the presence of platelet antagonists accounts for the “late” thrombin increase. The features of the second peak reflect the contribution of expressed PS to total TG. The appearance of the second peak in PRP-TGT demonstrates that the rate of PS expression on patient's platelets during their activation can be reduced. Taking this into account can help for more sensitive and precise antiplatelet therapy monitoring. For example, if the patient's PRP shows two peaks without any antiplatelet drugs, this could indicate that a lower dose of these drugs should be considered. This phenomenon can testify to a repression of platelet activation even if the ETP value is normal. According to our findings, the platelet concentration in PRP TGT should be the same for all the samples used in clinical and fundamental investigations. In spite of the independence of the ETP value on the concentration of platelets, the shape of the TGT strongly depends on it. If the platelet concentration is fixed, we can testify that appearance of two peaks in TGC is really linked with repression of platelet activation, but not to the low concentration of platelets. The modified TGT in the presence of a low concentration of PGE_1_ or with 1.6% DMSO can help to estimate the contributions of plasma phospholipids and PS-expressing platelets to the total thrombin generation in normal coagulation and different pathologies.

## Supporting Information

File S1 **Supporting Information Figures.**
(DOC)
