# Supplementary material for: Antiplatelet Agents Can Promote Two-Peaked Thrombin Generation in Platelet Rich Plasma: Mechanism and Possible Applications
Source: PLoS One. 2013 Feb 6;8(2):e55688. doi: 10.1371/journal.pone.0055688 (PMC3566002; doi:10.1371/journal.pone.0055688)
Supplement: File S1 — Supporting Information Figures. (DOC) [file pone.0055688.s001.doc]

**Supporting Information**


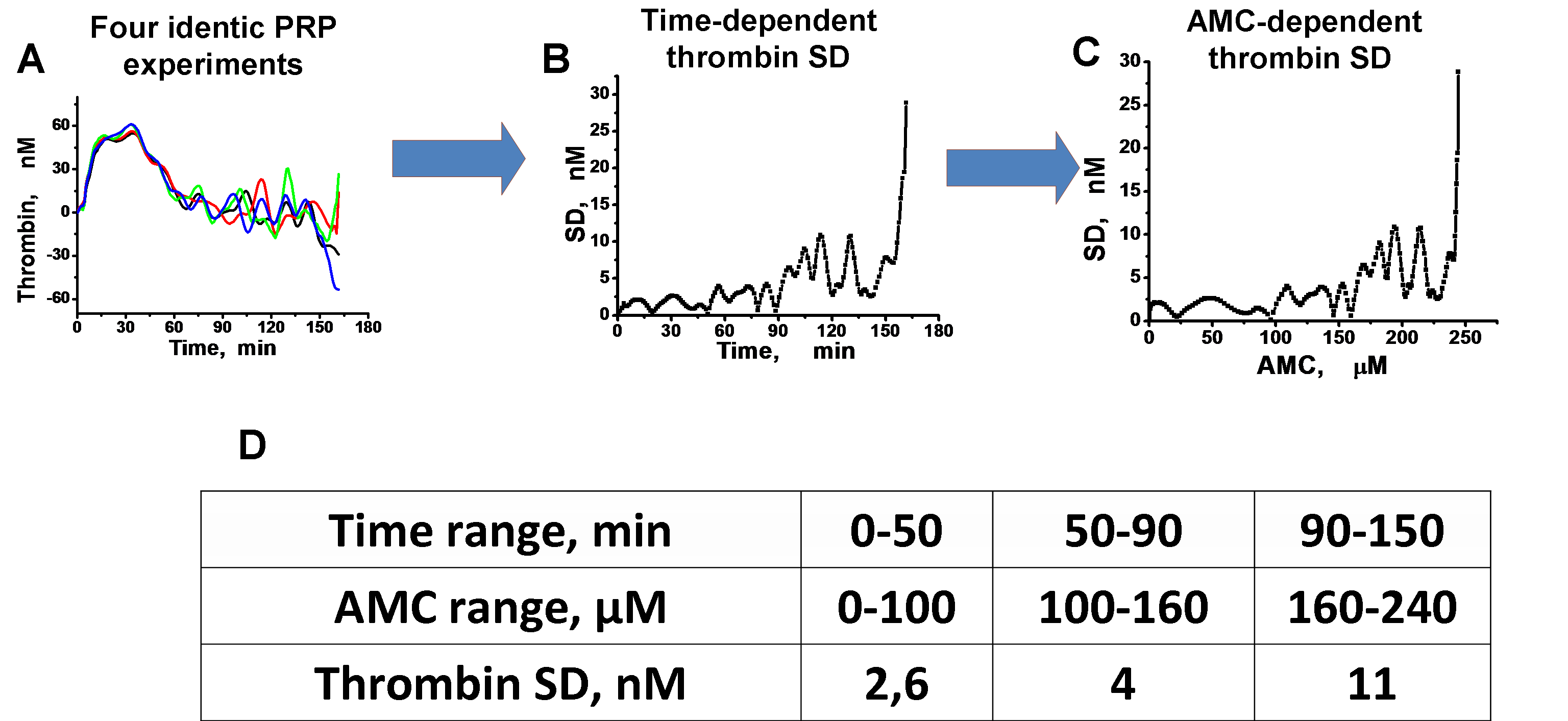


**Figure S1.** **Effect of AMC concentration on the standard deviation in the determination of thrombin concentration curve.** (A) Thrombin concentrations calculated in four repeated experiments with PRP from a single healthy donor. (B) The standard deviation (SD) for thrombin concentration calculated using data presented in (A). (C) SDof thrombin concentration as a function of AMC concentration in the sample. (D) The maximal SD values for thrombin generation experiments that are characteristic for different time and AMC concentration ranges.

**
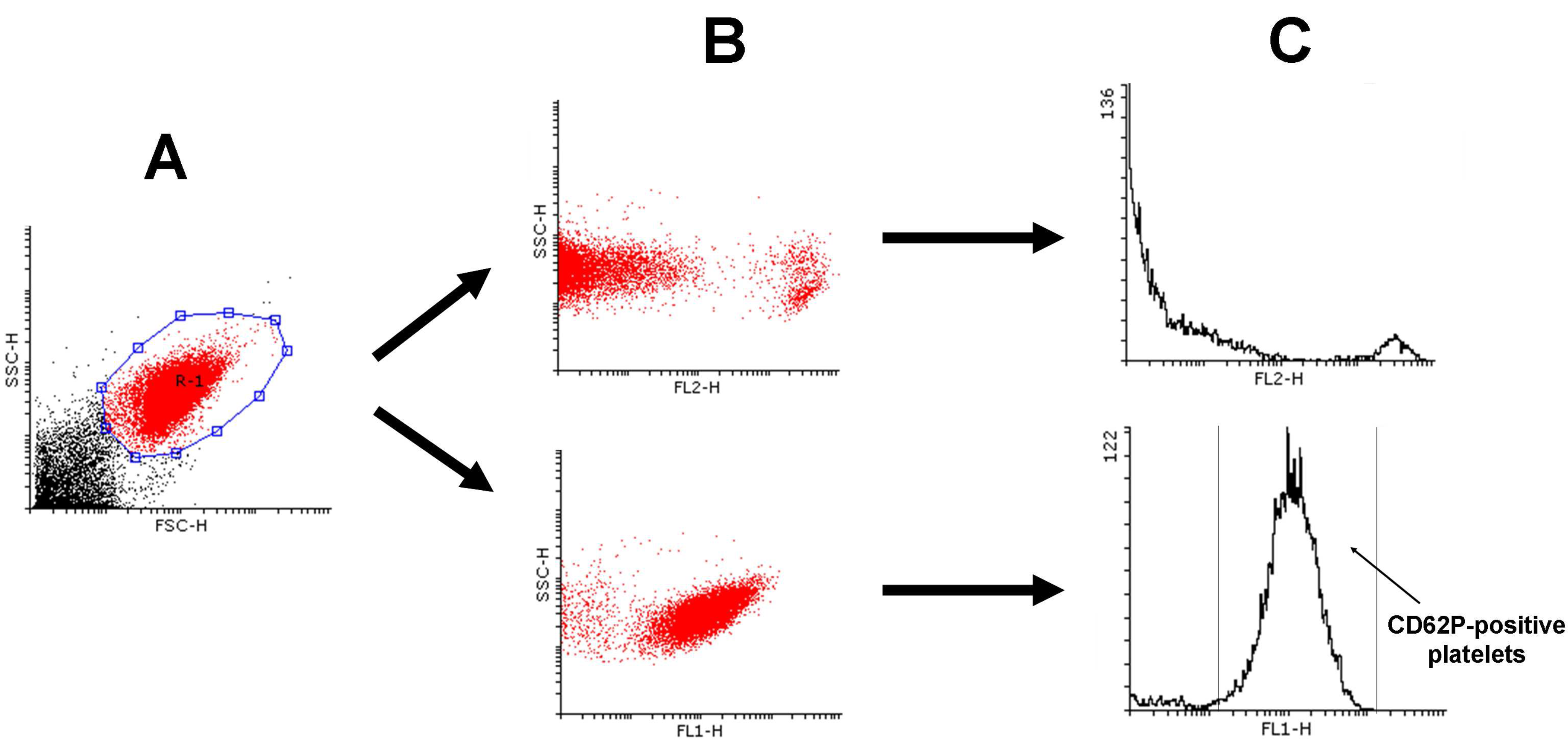
**

**Figure S2. Determination of mean fluorescence intensities of annexin V-RPE and anti-CD62P-FITC in a suspension of washed platelets.** Platelet suspension was activated as described in the section “Materials and Methods”, labeled with fluorescent antibodies, and subjected to flow cytometry. (A) Gating of the platelet region in the FSC-SSC dot plot. (B) Dot plots in the SSC-versus-fluorescence axes for each of the fluorescent labels, where FL1 is the anti-CD62P-FITC fluorescence channel and FL2 is the annexin-V-RPE fluorescence channel. (C) Histograms of annexin V-RPE and anti-CD62P-FITC fluorescence for calculation of mean annexin V fluorescence intensity and percentage of CD62P-positive platelets for the dot plots presented in (B).


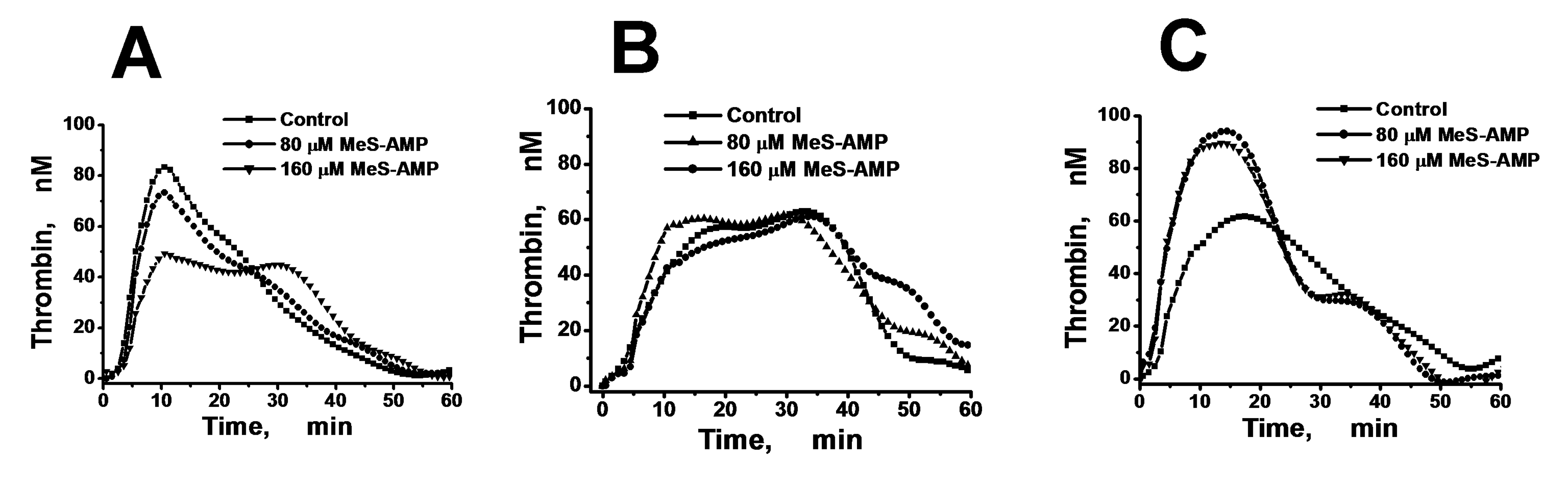


**Figure S3.** **Effect of 2-MeS-AMP on thrombin generation in different samples of PRP.** TGCs obtained for PRPs of three different healthy donors (A-C) are presented. Platelet concentration was equal to 100۰10^3^ platelets per µl, coagulation was induced with TF at 2 pM.


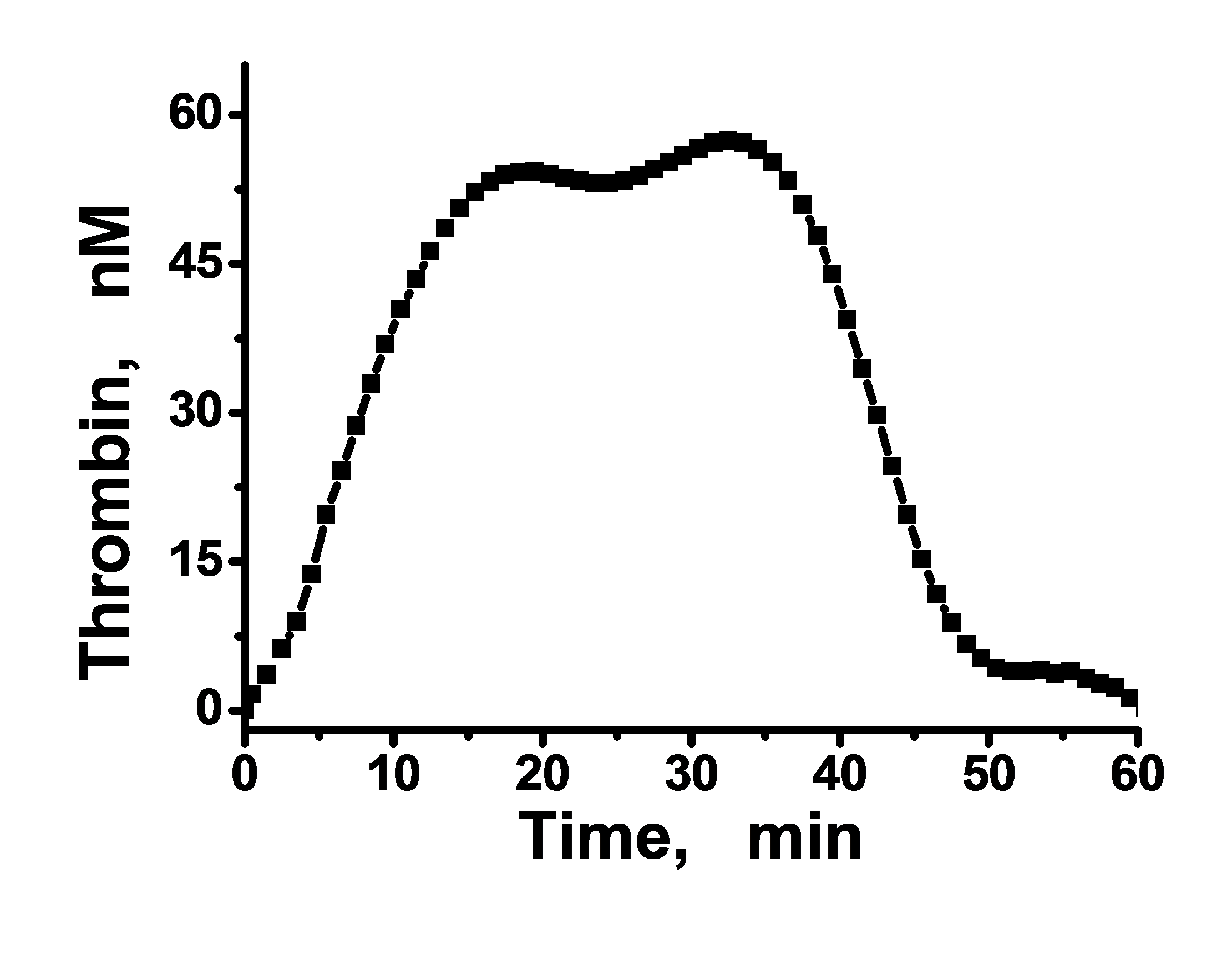


**Figure S4.** **Two-peaked PRP TGC in a sample containing 0.4% DMSO.** PRP from a healthy donor contained 100۰10^3^ platelets per µl, coagulation was triggered with TF at 2 pM.


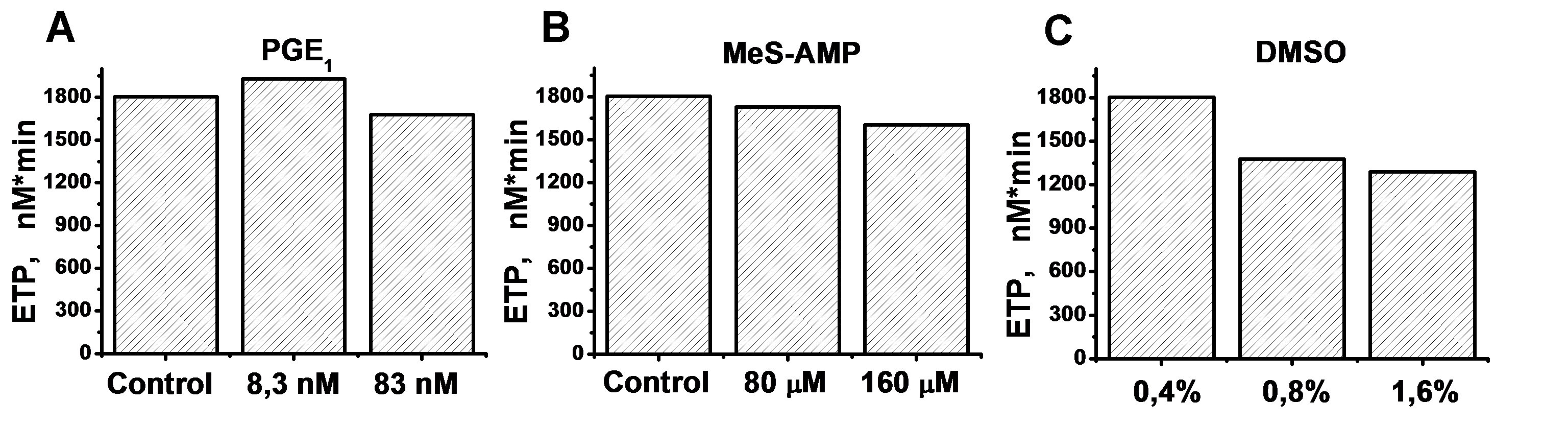


**Figure S5**. **PGE_1_ (A), MeS-AMP (B), and DMSO (C) weakly affect ETP in PRP.** Typical results for plasma of one donor are presented. Platelet concentration was equal to 100۰10^3^ µl^-1^ Coagulation was triggered with TF at 2 pM.


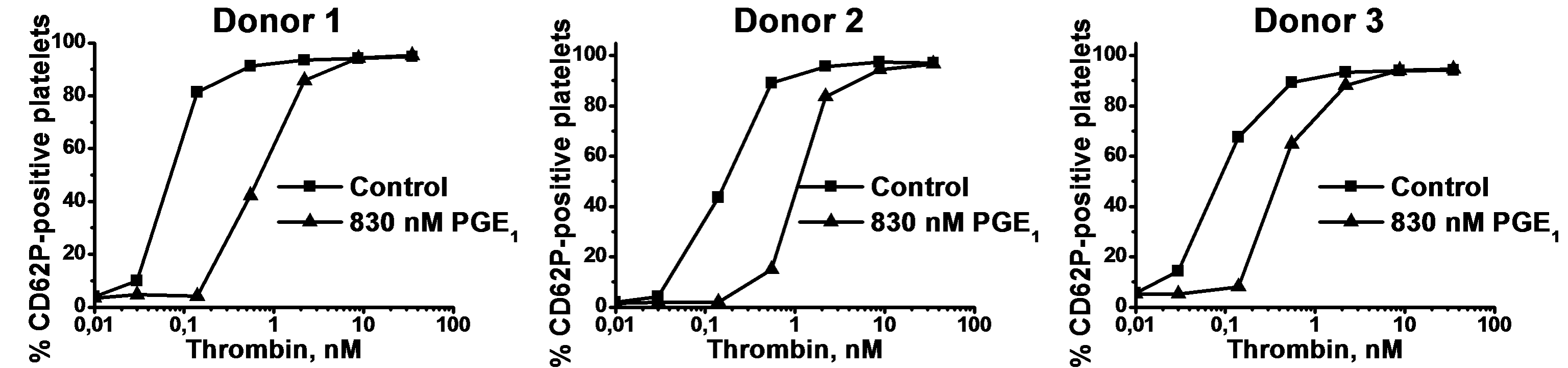


**Figure S6. Effect of PGE_1_ on CD62P release at different thrombin concentrations.** Suspension of washed platelets labeled with anti-CD62P-FITC were activated with solution containing different concentrations of thrombin for 5 min in the absence (control), or presence of 830 nM PGE_1_. After that, the percentage of CD62P-positive platelets was measured by flow cytometry. Final thrombin concentrations were 0.01 nM; 0.035 nM; 0.135 nM; 0.545 nM; 2.19 nM; 8.75 nM and 35 nM. Final platelet concentration was 100۰10^3^ platelets per 1 µl.


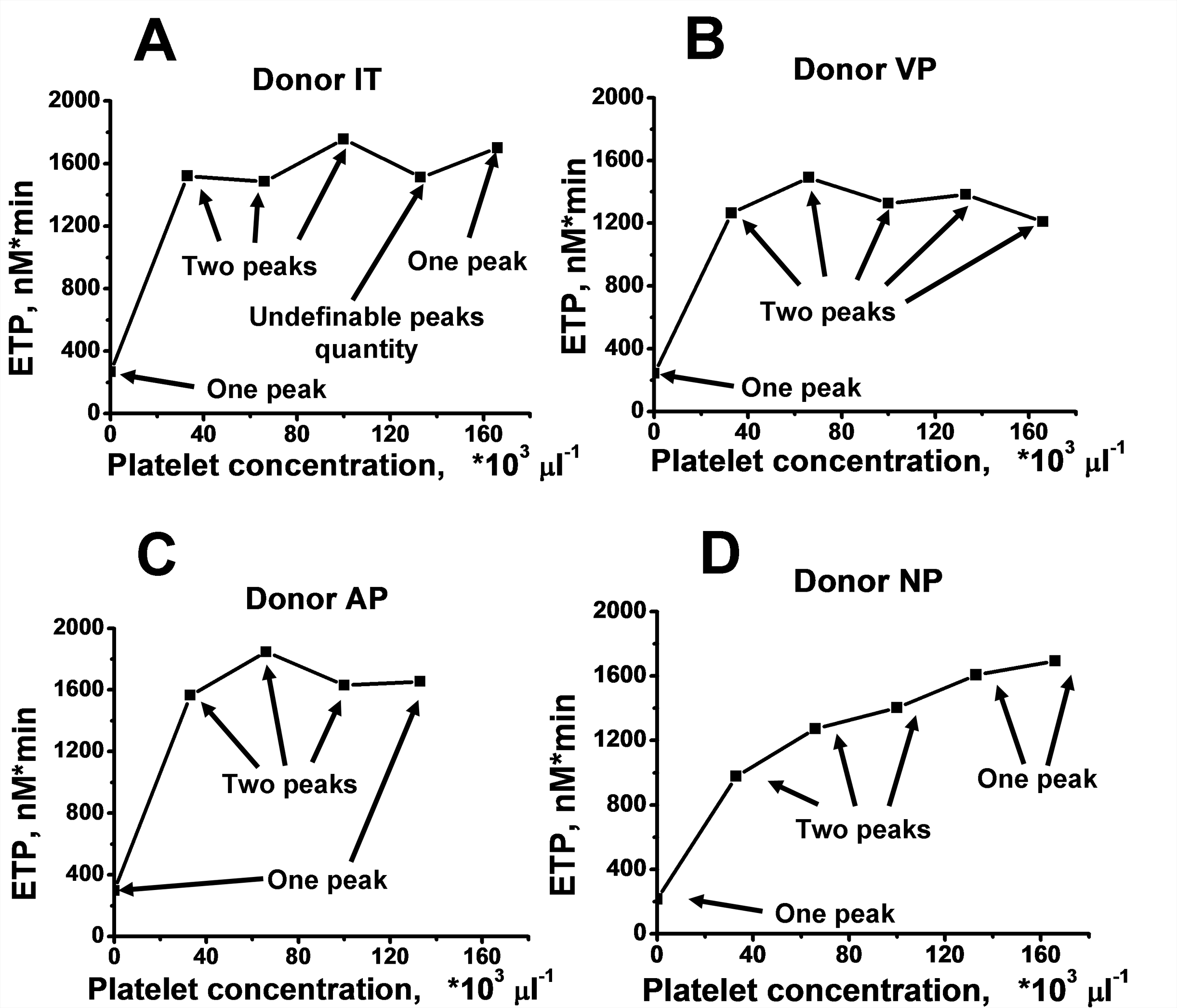


**Figure S7.** **Dependence of the ETP value and of the number of peaks on platelet concentration.** TG experiments were performed in plasma of healthy donors containing different concentrations of platelets as described in the section “Materials and Methods”. Results are presented for four different donors (A-D). All samples contained 1.6% DMSO. Coagulation was induced with TF at 2 pM.


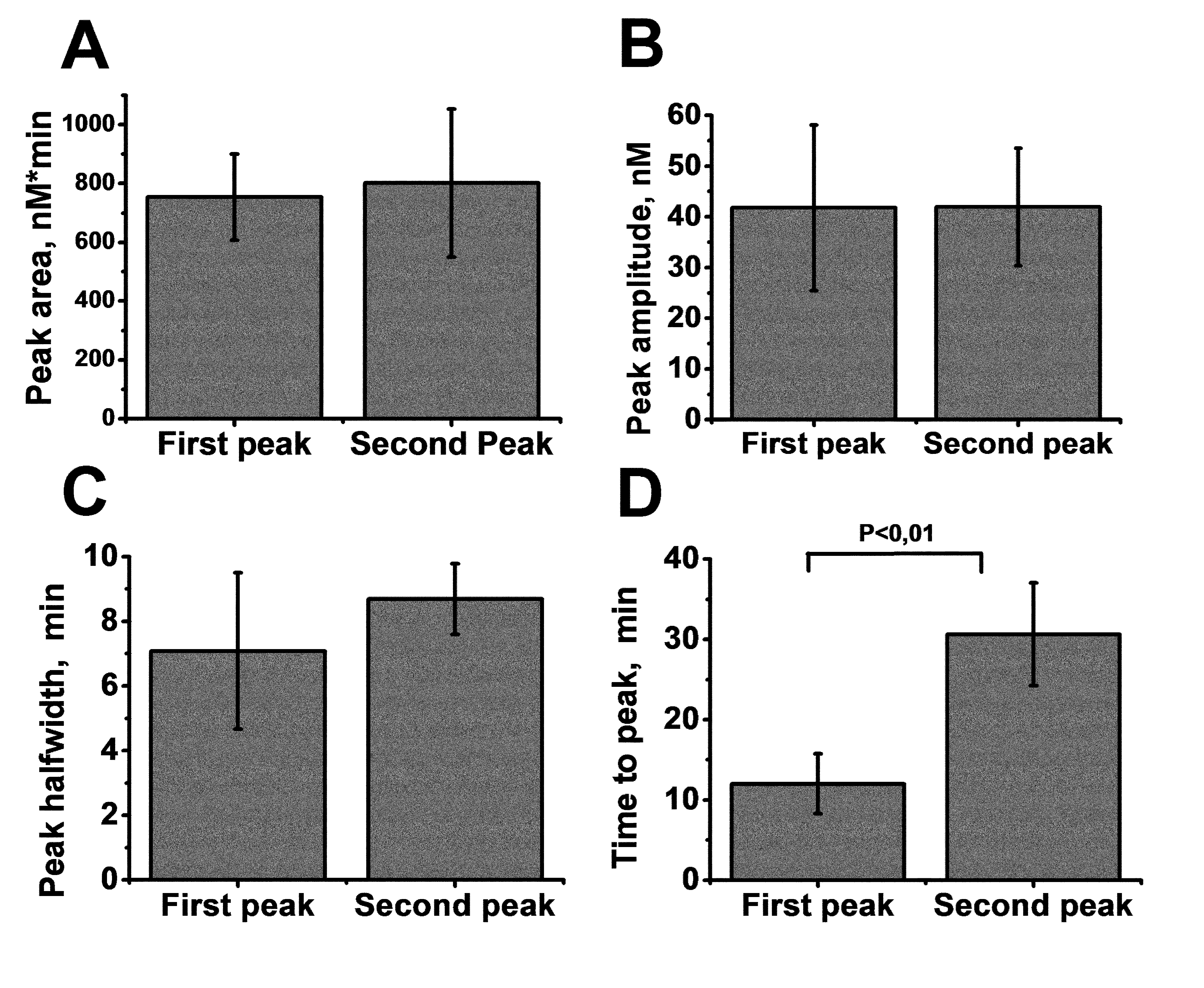


**Figure S8.** **Averaged thrombin generation parameters obtained for each peak of the two-peaked TGCs in PRP of healthy donors.** TG experiments were performed in plasmas of healthy donors containing different concentrations of platelets as described in the section “Materials and Methods”. Platelet concentration was equal to 100۰10^3^ µl^-1^, n=18. All the PRP samples contained 1.6% DMSO. Mean values and SD are presented for areas under the first and the second peaks (A), peak amplitudes (B), times to peaks (C), and half-widths for each peak (D). Coagulation was triggered with TF at 2 pM. The difference between peaks is significant only for the time to peaks (Student’s t-test, P<0.01).


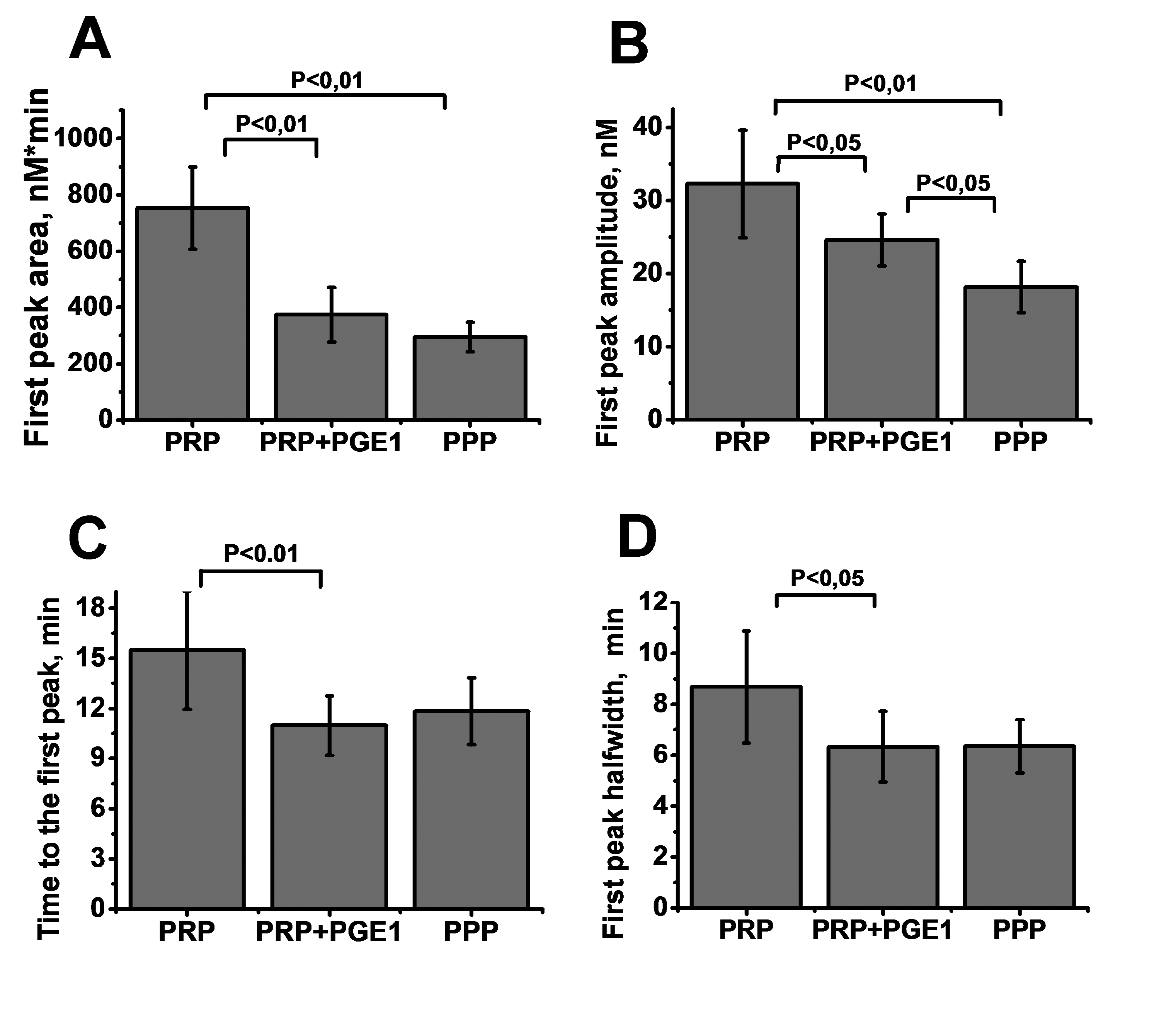


**Figure S9.** **Averaged parameters of the first peaks of thrombin generation curves obtained for PRP, PRP with 830 nM of PGE_1_ and PPP.** All samples contained 1.6% DMSO. Mean values and SD for area under the peak (A), peak amplitude (B), time to peak (C), and peak half-width (D) are presented. Results are shown for 18 TGCs measure in PRP, 8 TGCs measure in PRP with PGE_1_, and 7 РРР curves. Coagulation was activated with TF at 2 pM. Student’s t-test was used to obtain statistics. The difference is not significant in all the bars, where P-value is not presented.
